# Professional Demeanor

**Published:** 1899-08

**Authors:** R. B. Gentle


ARTICLE VI.

### PROFESSIONAL DEMEANOR.

R. B. GENTLE.

In writing on this time-honored subject, it is not my purpose to dwell at any length on the ordinary and natural requirements of a practitioner, but rather to bring to your notice a few points, which may not have had your especial attention.

There are conditions which arise in the practice of our profession which, if met as their peculiarity demands, will as a rule terminate to the advantage of the practitioner; while, on the other hand, if the practitioner is not equal to the occasion, the opportunity is lost, and notwithstanding the fact that he will probably pass the matter with the consideration that as nothing was at stake, nothing was lost, he has very likely lost something that would have been of great value.

> "There is a tide in the affairs of men.
> Which taken at the flood leads on to fortune;
> Omitted, all the voyages of their life
> Is bound in shallows and in miseries,"

If that were true in the time of Shakespeare it is no less true at the present time, and there are countless numbers of opportunities that pass by and we think lightly of them, until we see them grow into things of beauty and grandeur.

To take advantage of these opportunities as they come before us from time to time requires tact, refinement, experience and ability.

To be able to conduct one's self in such a calm, gentlemanly, yet stern manner, that an old maid having contempt for all men, after venting all her spleen on him by words and acts recognizes that she is not only unable to ruffle his temper, but is lowering herself in his estimation—or to be able to meet, with unflinching eye, the "know-all," who would kill you with a look, or the bluffer who would have you understand that you must do according to his dictations; I say to be able to do these things requires diplomacy or tact.

Refinement is a prerequisite to all in the upper walks of life, a thing easily acquired if the environments are conducive thereto, and in this age of advancement and competition, what profession or occupation requires refinement more than that of practicing dentistry? Personal appearance and cleanliness have much to do with the impressions made by different people when circumstances bring them in contact one with another.

Whether or not they realize it, nearly all people are impressionists, and it is the first impression formed that nearly always stands, whether it be right or wrong, as that intimacy necessary to the uprooting of a wrong impression is seldom allowed us—especially in this transient West.

We have heard it said that "The apparel oft proclaims the man," and while we do not sanction foppery, or extravagance, yet it is better to be a liitle over-dressed than sadly negligent in that regard. Lord Chesterfield in his plain English speaking way says, "One who is careless about his person at twenty will be a sloven at forty and filthy at fifty." I fear that we have in our ranks some who were careless at twenty. It is an easy matter for an operator to step back from the chair and fill his lungs with good, wholesome air, but not so with patients, and it is with reluctance that they will submit to a second seance where a foulness pervades the air.

The elevated position of our profession is maintained by the members individually.

That gentlemanly demeanor is just as essential in the office as it is in the parlor, and it is as easy to treat the less fortunate with courtesy as otherwise.

Should the laborer, dressed in his homespun, call to consult you regarding some work and the probable cost thereof, be gentlemanly and plain with him, as he may not be familiar with the technical terms used by you every day, and should you do his work, let there be no better done, because you are receiving hard-earned dollars from a representative of labor, the backbone and salt of the earth. He may leave your office thinking that your prices are exorbitant, but he cannot but think you are a gentleman. On the other hand, if his work is not the kind that you desire, recommend him to another dentist in a manner that his feelings may not be hurt. Some of these people are very sensitive and as we by our own choice have become servants of the people, we should at least treat them with common courtesy.

To know how and when to act on most occasions not only requires the power of reading human nature, but the practical experience of dealing with people from our professional side of the fence, and while I have spoken of maintaining our professional dignity, yet there are times when we are called upon to assert our rights and resent abuse. There does exist a class of people who cannot be handled with gloves, and as we cannot always get rid of them, they should be handled as their case demands. It was once my displeasure to see a man who sat down in a chair to have a tooth extracted, who told the dentist that if he broke his tooth he would knock him down. He was immediately dismissed. That was the only tooth I ever wanted to extract.

Directly in reverse of this class of patients we have the little folks to deal with, and if they have been lied to at home they will expect you to lie to them, and you have to go through the ordeal of restoring confidence in them, which can only be done by patience and honesty. If you can't devote to them the necessary time, give some less busy dentist a chance, and don't by your gruffness and impatience impress them with a feeling of horror for the dental office at a period of life when they need so much the care and advice of the dentist. The dentist who carries his frowns continually at the chair has no business with the little ones, to whom the first sitting means so much.

The ability to preserve that gentlemanly demeanor at all times depends largely on the state of one's health. It only takes about eight months' work at the chair to exhaust all the smiles that one is able to muster up. Then it is time to attend the meeting of some dental society and find out that life is really worth living. In this Western country where nature has endowed us with so many beautiful, lakes, rivers and mountains, to which we can go for recreation, there is little excuse for us having faces pinched and care-worn from confinement.

It is not conducive to mental peace to have too many irons in the fire at one time. No one man can do and be every thing.

"The man who seeks one thing in life and but one
May hope to achieve it before life be done,
But he who seeks all things wherever he goes
Only reaps from the hopes which around him he sows
A harvest of barren regrets."

We can't climb to any height on two ladders which point to different stars. There is no middle round in the ladder on which we can stop, if we wish to keep abreast with the profession. And as we advance along the line of new thought, let our demeanor be such that we may rank second to none in the eyes of the social and professional world.—"Pacific Medico-Dental Journal.

